# An examination of physical violence against women and its justification in development settings in Uganda

**DOI:** 10.1371/journal.pone.0255281

**Published:** 2021-09-29

**Authors:** Paul Bukuluki, Peter Kisaakye, Symon Peter Wandiembe, Tina Musuya, Evelyn Letiyo, Dan Bazira

**Affiliations:** 1 School of Social Sciences, Makerere University, Kampala, Uganda; 2 School of Statistics and Planning, Makerere University, Kampala, Uganda; 3 Centre for Domestic Violence Prevention, Mulago, Uganda; 4 UN Women, Kampala, Uganda; Shahjalal University of Science and Technology, BANGLADESH

## Abstract

This paper uses data from a community cross-sectional survey to examine the factors that are associated with justification of physical violence against women. Results indicate that respondents who were married at the time of the survey were less likely (OR = 0.29; CI = 0.17–0.52) to agree that it is justified for a man to physically assault his partner that their counterparts who were single. The likelihood to justify physical violence was less likely to happen among respondents with primary education (OR = 0.49; CI = 0.39–0.62), secondary education (OR = 0.40; CI = 0.31–0.53) and vocation or tertiary education (OR = 0.28; CI = 0.19–0.41) than among respondents with no education. Protestants were less likely (OR = 0.77; CI = 0.64–0.94) to justify physical violence than the Catholics. Respondents who were not formally employed were more likely (OR = 1.66; CI = 1.32–2.08) to justify physical violence than their counterparts who were in formal employment in the last three months preceding the survey. Respondents who agreed that it is okay for a man to control his partner’s movements (OR = 1.27; CI = 1.04–1.55), it is okay for a man to have sex with his wife anytime (OR = 2.28; CI = 1.87–2.78), alcohol is the main reason for violence against women (OR = 1.67; CI = 1.33–2.10), men need sex more than women (OR = 1.57; CI = 1.23–1.99) and women know where to obtain support in case of violence (OR = 1.42; CI = 1.00–2.02) were more likely to justify physical violence than respondents who disagreed. The likelihood to justify physical violence was less among respondents who agreed that: violence is not the only way to deal with disagreements (OR = 0.54; CI = 0.33–0.86), it is possible for men to stop violence (OR = 0.62; CI = 0.47–0.82) and it is acceptable for a woman to ask her partner to use a condom (OR = 0.61; CI = 0.51–0.73) than their counterparts who disagreed. There is need to increase investment in social norms change programmes in order to strengthen contestation of tolerance of physical violence among men and women in Uganda.

## Introduction

Experience of physical violence is a human rights abuse issue with negative consequences [1]. Physical violence refers to intentionally using exercising physical aggression to cause physical harm or death [2]. Physical violence includes beating, biting, kicking, slapping or strangling someone [3]. Globally, about a third (30%) of women have ever experienced a form of physical violence [4]. Experience of physical violence is reported to be highest in developing countries, including in sub-Saharan Africa (SSA) [5,6]. For example, about 36% of women in SSA have experienced some form of intimate partner violence [6–8]. Moreover, women are disproportionately likely to experience physical intimate partner violence compared to men [9–12]. Physical violence remains high in SSA because it is justified in some societies [13]. In this paper, we define justification of physical violence as psychological or social processes based on context specific reasons such as social and gender norms that lead to a partner to view physical violence as normal [14].

The government of Uganda has enacted a number of policies and measures that address experience of violence. For example, the constitution of Uganda under Article 33 provides for equal dignity of women, and prohibits any form of law or patriarchal system that undermines women’s dignity [15]. In addition, the National Policy on the Elimination of Gender Based Violence that was developed in 2016 to encourage stakeholders to increase and expand their programmatic efforts in preventing and responding to gender based violence (GBV) [16]. The Uganda Gender Policy that was enacted in 2007 to provide a framework for identification, implementation and design of interventions aimed at promoting gender equality and women’s empowerment [17]. The National Referral Pathway for Prevention and Response to GBV Cases in Uganda (2013) aims to provide assistance to victims or survivors of GBV [18]. Despite the measures taken by the government of Uganda to address the issue of violence, experience of violence against women remains high. That is, results from a nationally representative survey indicates that about half of Ugandan women (51%) aged 15 years and older have ever experienced physical violence [19].

One reason violence against women persists is because some people either justify or tolerate it [20]. A recent study reports that tolerance of violence is likely to be higher among people of low-socio-economic status, less education [21] and unemployed people [22,23]. Socially, violence may be acceptable for many reasons including social norms [24], support for patriarchy or male dominance [25] or controlling behaviors [26,27]. In addition, violence is more likely to occur in relationships that have a prior incident of violence [28], unequal power sharing [29], being poor [30,31], or a past experience of conflict in couples or families [32]. Intimate partner violence (IPV) is a reflection of unequal gender relations that allow for male dominance. Scholars have conceptualized this as hegemonic masculinity.

Hegemonic masculinity is describes how gender, power and oppression are embodied in relationships between men and women [33]. Many descriptions of hegemonic masculinity exist; all point to the notion that it involves a specific strategy for the subordination of women [33–36]. It has been argued that hegemonic masculinity contributes to sustaining hierarchical gender relations between men and women, allowing such relations to be perceived as normal and legitimate [24,33]. Connell and Messerschmidt (2005) further argue that hegemonic masculinity reflects a pattern of a relationship and legitimates unequal gender relations in context-specific ways [36].

Hegemonic masculinity operates through social and gender norms; such norms are the informal rules which govern the behavior of individuals in specific cultural contexts [37–39]. In this context, they normalize the subordination of women. Hegemonic masculinity may contribute to the tolerance of unequal gender relations and the acceptability of violence against women [37,40]. In addition, structural level factors can support tolerance of violence against women and female subordination. Some authors have argued that positive masculinity is an alternative to hegemonic masculinity. Positive masculinity supports alternative norms and behaviors that are opposed to gender inequality and the subordination of women [24,38,41].

Schippers, (2007) argues that hegemonic femininity represents an ideal picture of womanhood that is aligned with gender norms including the perception that women should be sweet, modest, pay attention to physical appearance and sexual attractiveness. Schippers further proposes that “hegemonic femininity consists of the characteristics defined as womanly that establish and legitimate a hierarchical and complementary relationship to hegemonic masculinity and that, by doing so, guarantee the dominant position of men and the subordination of women”[42]. This hegemonic femininity is relative and may manifest differently in various societies. It complements the subordination of women thereby further entrenching unequal gender relations used to justify and normalize gender based violence in various societies [43].

In Uganda, previous research has reported a higher experience of IPV among women who have less education [44,45], HIV [46], do not consent to having sex with their partner [47], are in a polygamous relationship [48], consume alcohol [49], have previously experienced conflict or war [50,51] or live in rural areas [52]. Despite a large body of evidence on the role of structural factors influencing violence against women, there is limited research of the factors that justify violence against women [53]. Most studies have focused on IPV in developed countries; few studies in developing countries have included the SSA region [5]. In this research, we aimed to investigate the factors justify physical violence against women in six Ugandan districts. Based on the literature, we hypothesize that acceptance of any controlling behavior as normal or having minimal reproductive rights as a woman is more likely to be associated with justification of physical violence [54]. The findings from this study can be used to support the design of interventions that aim to reduce or end social tolerance of physical IPV in Uganda.

## Data and methods

### Study design and methods

The data used in this study come from a community cross-sectional survey that collected quantitative data to establish a baseline for projects that were going to address IPV using the SASA! Model. SASA! which stands for Start, Awareness, Support and Action is a community mobilization strategy that is used to prevent violence against women [55,56]. A detailed explanation of the SASA! is found elsewhere [4,55–63].

### Study sites and population

The research study was conducted in six districts of Uganda (Amudat, Kaberamaido, Kasese, Moroto, Tororo and Pader) where the EU Spotlight Initiative to eliminate violence against women and girls is implemented [64,65].

### Community sample survey, design and size

The community survey followed a stratified two-stage cluster design, with districts as study domains. In the first stage, a simple random sample of ten villages was taken using probability proportional to size (approximate number of households) sampling in each district. In the second stage, lists of locations within the selected villages were classified into small and large areas or routes or path junctions of traffic. A list of locations within each district was developed together with community leaders. A simple random sample of 3–5 small locations and 2–3 large locations were chosen. Finally, a systematic random sampling approach was used to recruit respondents for interview. A systematic random sample was used as it is easy to implement and has high internal and external validity [66–68]. A total of 3456 individuals were identified and approached for interview. Of these, 3427 (99%) participated in the study.

### Data collection

Data was collected using a quantitative community survey tool by a team of four well trained research assistants per district (two females and two males) in August-September 2019. Interviews were done in a private setting. Responses were electronically recorded using a mobile tablet programmed with a survey tool. The data was synchronized on a daily basis onto an online server managed by a research consultant. Due to the sensitive nature of some of the questions, female interviewers would interview only female respondents and male interviewers interviewed male respondents. Further, research assistants were trained to have some empathy and make arrangements for referrals to service providers who can offer psychosocial support should they observe any signs of tremor. Prior to the actual data collection, a pre-test was done to test the validity and reliability of the questionnaire and lessons learned were incorporated in the final revision of the questionnaire.

### Variables

Socio-economic and demographic variables include sex (female or male), age in single years, current marital status (single, currently married, widowed or separated or divorced), education (no education, primary, secondary, vocational or university), religion (Catholic, Protestant, Born again Christian, Muslim, Seventh Day Adventist (SDA), Traditional) and formal employment in last three months preceding the survey (no or yes).

The study also collected information on people’s attitudes towards violence against women (it is okay for a man to control his wife’s movements, it is okay for a man to have sex with his wife anytime, men’s power is the reason for violence against women, violence is not the only way to deal with disagreements, it is possible for men to stop violence, alcohol is the main reason for violence against women and girls, it is acceptable for a woman to ask her partner to use a condom, a woman can decide the number of children to have and when to have them, men need sex more than women and women are raped because of the way they dress). Responses to each of these attitudinal questions were disagree or agree. Women were also asked whether they know where to obtain support in case of violence; those who said yes were asked to describe where women could seek support: legal, shelter, medical and psychosocial services.

Respondents were asked whether it is justified for a man to hit his partner when she burns food, argues with him, goes out with permission, neglects their children, refuses sex or uses contraception without his knowledge. Respondents who agreed with at least one justification for violence against women were considered accepting of violence against women.

### Data analysis

Using STATA, frequencies were calculated for each variable. A Chi-square test was used to assess bivariate relationships between selected variables and justification of physical violence against women. At the multivariate level of analysis, a binary logistic regression model was fitted to examine the determinants of justification of physical violence against women. Before running the regression model, a collinearity test that shows whether the variables included in the model are independent from each other was performed and, results pointed to no or minimal correlation between variables as the estimated tolerance value–a collinearity diagnostic measure–was close to one for all variables in the study.

### Ethical considerations

This study was approved by the Research and Ethics committee at the School of Social Sciences, Makerere University. Verbal informed consent was obtained from each respondent before the interview. During the process of getting informed consent, respondents were assured of safety, confidentiality, voluntary participation and were at liberty to respond to questions they were comfortable with or face no consequences for withdrawing from the study.

## Results

The majority of respondents were female (66%), currently married (80%), and had completed primary education (43%) (Table 1). Half of the respondents (50%) belonged to the Catholic religious faith. Most respondents (85%) reported that they were not in formal employment in the last three months preceding the survey.

**Table 1:** Distribution of respondents by socio-economic and demographic factors.

Variable	Number	Percent
Sex		
Male	1159	33.8
Female	2268	66.2
Age of respondent		
15–19	349	10.2
20–24	576	16.8
25–29	674	19.7
30–34	558	16.3
35–39	462	13.5
40–44	349	10.2
45+	459	13.4
Current marital status		
Single	466	13.6
Married	2740	79.9
Widowed/Divorced/Separated	221	6.5
Education		
No education	1005	29.3
Primary	1491	43.5
Secondary	707	20.6
Vocational/University	224	6.5
Religion		
Catholic	1735	50.6
Born again	556	16.2
Protestant	994	29
Traditional	51	1.5
Moslem	54	1.6
SDA	37	1.1
Formally employed in last 3 months		
No	2904	84.7
Yes	523	15.3
Total	3427	100

Note: SDA = Seventh Day Adventist. Missing cases are not shown. Figures may not add up to 100 percent due to rounding errors.

Table 2 presents findings from the bivariate analysis comparing explanations to justify violence against women by men and women participating in the study. Men and women reported significant differences in their agreement with reasons to justify violence against women including whether a man should control his wife’s movements (p<0.01), is allowed to have sex with his wife anytime (p<0.01), men’s power is the reason for violence against women (p<0.001), it is possible for men to stop violence (p<0.001), alcohol is the main reason for violence against women and girls (p<0.001), a woman can decide the number of children and when to have them (p<0.001), men need sex more than women (p<0.001), women are raped because of the way they dress (p<0.05) and women know where to obtain support in case of violence (p<0.05).

**Table 2:** Distribution of respondents by gender and attitudes towards violence against women.

Variable	Male	Female	Chi-square (P-value)
It is okay a man to control his wife’s movements			8.326 (0.004)***
Disagree	37.7	62.3	
Agree	32.4	67.6	
It is okay for a man to have sex with his wife anytime			8.580 (0.003)***
Disagree	35.9	64.1	
Agree	31.1	68.9	
Men’s power is the reason for violence against women			149.463 (0.000)****
Disagree	56.0	44.0	
Agree	29.4	70.6	
Violence is not the only way to deal with disagreements			3.620 (0.057)
Disagree	40.3	59.7	
Agree	33.5	66.5	
It is possible for men to stop violence			30.738 (0.000)****
Disagree	22.1	77.9	
Agree	35.5	64.5	
Alcohol is the main reason for violence against women and girls			34.678 (0.000)****
Disagree	23.3	76.7	
Agree	36.0	64.0	
It is acceptable for a woman to ask her partner to use a condom			1.230 (0.267)
Disagree	34.7	65.3	
Agree	32.9	67.1	
A woman can decide the number of children and when to have them			23.687 (0.000)****
Disagree	36.7	63.3	
Agree	28.4	71.6	
Men need sex more than women			49.373 (0.000)****
Disagree	48.6	51.4	
Agree	31.7	68.3	
Women are raped because of the way they dress			5.441 (0.020)**
Disagree	30.2	69.8	
Agree	34.8	65.2	
Women know where to obtain support in case of violence			5.018 (0.025)**
No	40.4	59.6	
Yes	33.3	66.7	
Total (N)	1159	2268	

Note:

** = p<0.05

*** = p<0.01

**** = p<0.001. Missing cases are not shown. Figures may not add up to 100 percent due to rounding errors.

Results indicate that males constituted the highest proportion (56%) of respondents that disagreed that men’s power is the reason for violence against women while females constituted the highest proportion (71%) of respondents that agreed that men’s power is the reason for violence against women.

### Bivariate relationship between selected factors and justification of physical violence against women

Table 3 indicates that the respondents were significantly different in terms of justification of physical violence by the age of the respondent (p<0.01), current marital status (p<0.001), education (p<0.001), religion (p<0.05) and formal employment in last three months (p<0.001). The results shown in Table 3 indicate that irrespective of any socio-economic and demographic factors, the highest proportion of respondents (77%) agreed that it is justified for a man to physically assault his partner.

**Table 3:** Relationship between socio-economic and demographic factors and justification of physical violence against women.

	Justification of physical violence		Chi-square (P-value)	Total
Variable	No	Yes		
Sex			0.3 (0.581)	
Male	23.6	76.4		100
Female	22.7	77.3		100
Age of respondent			19.6 (0.003)***	
15–19	12.2	87.8		100
20–24	21.6	78.4		100
25–29	23.7	76.3		100
30–34	25.1	74.9		100
35–39	23.5	76.5		100
40–44	24.0	76.0		100
45+	25.5	74.6		100
Current marital status			102.1 (0.000)****	
Single	0.0	100.0		100
Married	25.8	74.2		100
Widowed/Divorced/Separated	9.3	90.7		100
Education			65.4 (0.000)****	
No education	15.0	85.0		100
Primary	25.1	75.0		100
Secondary	26.0	74.1		100
Vocational/University	37.9	62.1		100
Religion			11.5 (0.042)**	
Catholic	21.8	78.2		100
Born again	21.3	78.7		100
Protestant	26.3	73.7		100
Traditional	12.0	88.0		100
Moslem	27.1	72.9		100
SDA	22.9	77.1		100
Formally employed in last 3 months			50.4 (0.000)****	
No	20.7	79.3		100
Yes	35.3	64.7		100
Total	23.0	77.0		100

Note:

** = p<0.05

*** = p<0.01

**** = p<0.001. Missing cases are not shown. Figures may not add up to 100 percent due to rounding errors. SDA = Seventh Day Adventist.

The results in Table 4 show that respondents were significantly different by a number of attitudinal statements in terms of justification of physical violence: it is okay for a man to control his wife’s movements (p<0.001), it is okay for a man to have sex with his wife anytime (p<0.001), violence is not the only way to deal with disagreements (p<0.01), it is possible for men to stop violence (p<0.05), alcohol is the main reason for violence against women and girls (p<0.05), it is acceptable for a woman to ask her partner to use a condom (p<0.001) and men need sex more than women (p<0.001).

**Table 4:** Relationship between attitudes towards violence and justification of physical violence against women.

	Justification of physical violence		Chi-square (P-value)	Total
Variable	No	Yes		
It is okay a man to control his wife’s movements			12.5 (0.000)****	
Disagree	27.4	72.6		100
Agree	21.4	78.6		100
It is okay for a man to have sex with his wife anytime			77.7 (0.000)****	
Disagree	28.9	71.1		100
Agree	15.6	84.4		100
Men’s power is the reason for violence against women			1.0 (0.309)	
Disagree	21.3	78.7		100
Agree	23.3	76.7		100
Violence is not the only way to deal with disagreements			8.4 (0.004)***	
Disagree	14.0	86.1		100
Agree	23.5	76.5		100
It is possible for men to stop violence			4.9 (0.027)**	
Disagree	18.7	81.3		100
Agree	23.6	76.4		100
Alcohol is the main reason for violence against women and girls			6.1 (0.014)**	
Disagree	27.1	73.0		100
Agree	22.1	77.9		100
It is acceptable for a woman to ask her partner to use a condom			37.6 (0.000)****	
Disagree	18.7	81.3		100
Agree	27.9	72.1		100
A woman can decide the number of children and when to have them			0.0 (0.922)	
Disagree	23.0	77.0		100
Agree	22.9	77.1		100
Men need sex more than women			17.1 (0.000)****	
Disagree	31.2	68.8		100
Agree	21.9	78.1		100
Women are raped because of the way they dress			0.0 (0.956)	
Disagree	22.9	77.1		100
Agree	23.0	77.0		100
Women know where to obtain support in case of violence			0.0 (0.999)	
No	23.0	77.0		100
Yes	23.0	77.0		100
Total	23.0	77.0		100

Note:

** = p<0.05

*** = p<0.01

**** = p<0.001. Missing cases are not shown. Figures may not add up to 100 percent due to rounding errors.

### Factors associated with justification of physical violence against women

Table 5 shows results from three multivariate models looking at different factors that explain male and female justification for violence against women. Model 1 only controls for socio-economic and demographic factors, Model 2 controls for only attitudinal factors and Model 3 controls for all factors (socio-economic, demographic and attitudes). The results in Model 1 indicate that marital status, education, religion and formal employment are related to justifying violence against women. That is, respondents who were married at the time of the survey were less likely (OR = 0.29; CI = 0.17–0.52) to agree that it is justified for a man to physically assault his partner than those were single at the time of the study. Further, holding justification of physical violence against women was less likely among those who completed primary education (OR = 0.49; CI = 0.39–0.62), secondary education (OR = 0.40; CI = 0.31–0.53) and vocation or tertiary education (OR = 0.28; CI = 0.19–0.41) than among respondents with no education. Protestants were less likely (OR = 0.77; CI = 0.64–0.94) to justify physical violence than the Catholics. However, Table 5 shows that respondents who were not formally employed were more likely (OR = 1.66; CI = 1.32–2.08) to justify physical violence than their counterparts who were in formal employment in the last three months preceding the survey.

**Table 5:** Factors associated with justification of physical violence against women.

	Odds Ratio (95%CI)		
Variable	Model 1	Model 2	Model 3
**Sex (RC = Female)**			
Male	1.05 (0.87–1.28)		1.10 (0.89–1.36)
**Age of respondent (RC = 15–19)**			
20–24	1.19 (0.73–1.94)		1.26 (0.75–2.11)
25–29	1.13 (0.70–1.82)		1.19 (0.72–1.96)
30–34	1.04 (0.64–1.69)		1.11 (0.67–1.85)
35–39	1.14 (0.70–1.87)		1.21 (0.72–2.03)
40–44	1.03 (0.62–1.73)		1.10 (0.64–1.88)
45+	0.98 (0.59–1.60)		0.93 (0.55–1.57)
**Current marital status (RC = Single)**			
Married	0.29**** (0.17–0.52)		0.31**** (0.18–0.55)
Widowed/Divorced/Separated	-		-
**Education (RC = No education)**			
Primary	0.49**** (0.39–0.62)		0.60**** (0.46–0.78)
Secondary	0.40**** (0.31–0.53)		0.52**** (0.38–0.72)
Vocational/University	0.28**** (0.19–0.41)		0.49*** (0.32–0.75)
**Religion (RC = Catholic)**			
Born again	0.86 (0.67–1.11)		0.81 (0.62–1.06)
Protestant	0.77** (0.64–0.94)		0.73*** (0.59–0.90)
Traditional	1.21 (0.50–2.94)		1.09 (0.43–2.70)
Moslem	0.85 (0.44–1.68)		1.08 (0.51–2.31)
SDA	1.14 (0.49–2.65)		1.19 (0.50–2.83)
**Formally employed in last 3 months (RC = Yes)**			
No	1.66**** (1.32–2.08)		1.86**** (1.47–2.37)
**It is okay a man to control his wife’s movements (RC = Disagree)**			
Agree		1.27** (1.04–1.55)	1.26** (1.02–1.55)
**It is okay for a man to have sex with his wife anytime (RC = Disagree)**			
Agree		2.28**** (1.87–2.78)	2.20**** (1.78–2.71)
**Men’s power is the reason for violence against women (RC = Disagree)**			
Agree		0.86 (0.68–1.10)	0.88 (0.68–1.14)
**Violence is not the only way to deal with disagreements (RC = Disagree)**			
Agree		0.54*** (0.33–0.86)	0.53** (0.33–0.87)
**It is possible for men to stop violence (RC = Disagree)**			
Agree		0.62*** (0.47–0.82)	0.63*** (0.47–0.85)
**Alcohol is the main reason for violence against women and girls (RC = Disagree)**			
Agree		1.67**** (1.33–2.10)	1.81**** (1.43–2.31)
**It is acceptable for a woman to ask her partner to use a condom (RC = Disagree)**			
Agree		0.61**** (0.51–0.73)	0.65**** (0.53–0.80)
**A woman can decide the number of children and when to have them (RC = Disagree)**			
Agree		0.92 (0.76–1.11)	0.91 (0.74–1.11)
**Men need sex more than women (RC = Disagree)**			
Agree		1.57**** (1.23–1.99)	1.54*** (1.19–1.99)
**Women are raped because of the way they dress (RC = Disagree)**			
Agree		0.99 (0.80–1.24)	1.07 (0.86–1.35)
**Women know where to obtain support in case of violence (RC = No)**			
Yes		1.42 (1.00–2.02)	1.45 (1.00–2.10)
Constant	11.68**** (5.40–25.27)	2.71*** (1.33–5.51)	5.63*** (1.91–16.58)

Note:

** = p<0.05

*** = p<0.01

**** = p<0.001. Reference Category (RC) in parenthesis. SDA = Seventh Day Adventist.

The results from Model 2 indicate that respondents who agreed that it is okay for a man to control his partner’s movements (OR = 1.27; CI = 1.04–1.55), it is okay for a man to have sex with his wife anytime (OR = 2.28; CI = 1.87–2.78), alcohol is the main reason for violence against women (OR = 1.67; CI = 1.33–2.10) and men need sex more than women (OR = 1.57; CI = 1.23–1.99) were more likely to justify physical violence than respondents who disagreed. On the other hand, the results in Table 5 show that the likelihood to justify physical violence was less among respondents who agreed that: violence is not the only way to deal with disagreements (OR = 0.54; CI = 0.33–0.86), it is possible for men to stop violence (OR = 0.62; CI = 0.47–0.82) and it acceptable for a woman to ask her partner to use a condom (OR = 0.61; CI = 0.51–0.73) than those who disagreed with the statements. The results presented in Table 5 indicate that after controlling for all socio demographic and attitudinal factors (Model 3), the pattern and direction of results remained the same as presented in Models 1 and 2.

**Fig 1:**
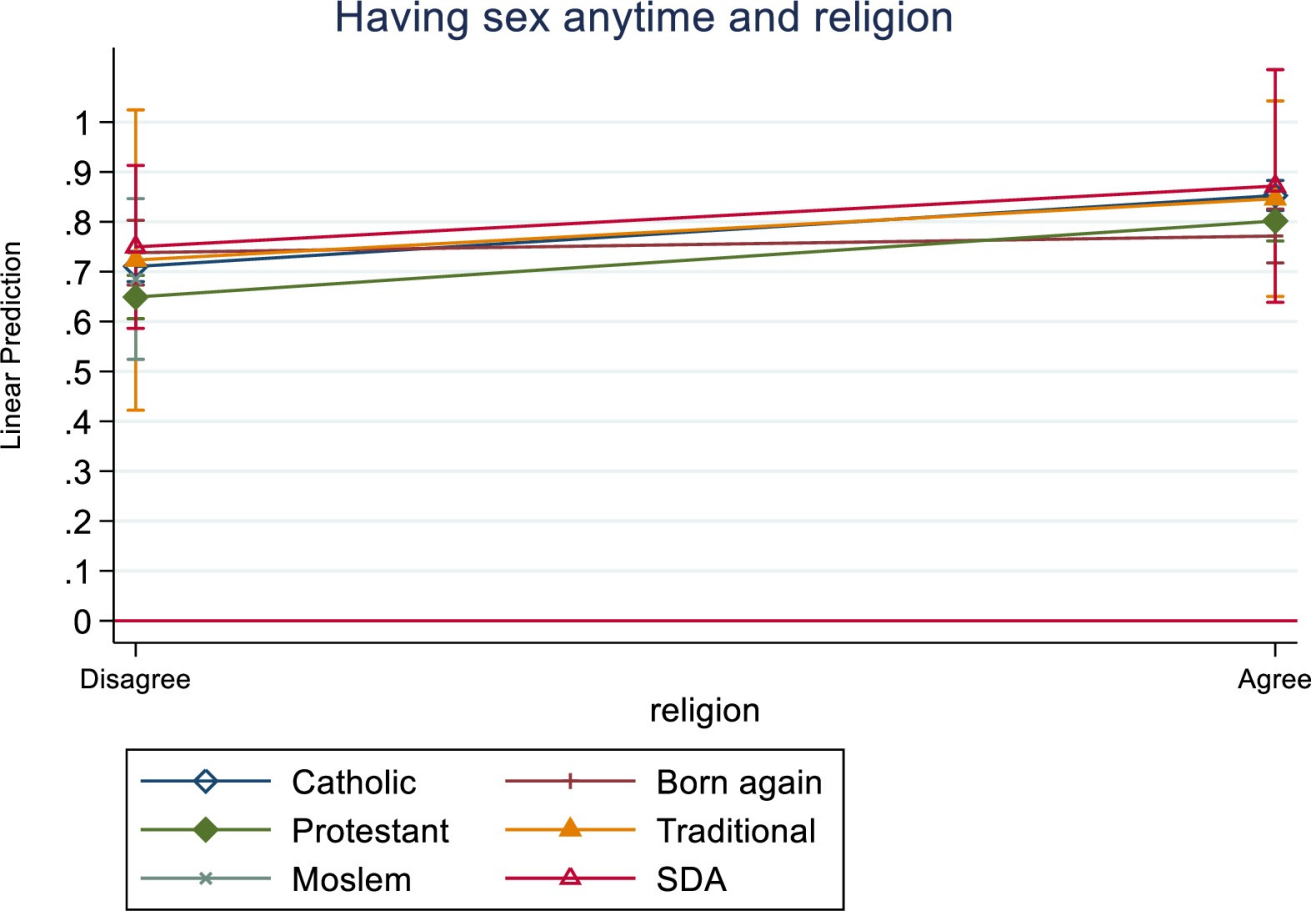
Marginal effects of a man having sex anytime with his wife and religion on justification of physical violence against women.

For further analysis, we included an interaction term in the model: it is okay for a man to have sex with his wife anytime (as a measure of reproductive rights) and religious belief to assess the effect of justification of physical violence against women (see Fig 1). This analysis was done on an assumption that responses about reproductive rights may be rooted in religious beliefs [69–71]. The results in Fig 1 show that irrespective of one’s religious belief, the likelihood to justify physical violence is higher for respondents who agreed that it is okay for a man to have sex with his wife anytime than those that disagreed. This finding is similar to the results presented in Table 5 –implying that while reproductive rights may be rooted in religious beliefs, there are other factors at play (beyond religious beliefs) that could influence the interrelationship between some reproductive rights and justification of physical violence.

## Discussion

This paper demonstrates the multiple factors that justify violence against women, including the socio-demographic and attitudinal factors that support physical violence against women [20,24,41]. The study describes several socio demographic factors that are associated with justification of violence against women including marital status, employment status and educational attainment [23,44,45,72]. For example, those who were married at the time of the survey reported less support for tolerance of violence against women than those who were single at the time of survey.

This is in line with the findings of Uthman and colleagues who found that respondents who were currently married from Malawi, Namibia, Rwanda, and Zimbabwe were less likely to justify IPV than those never married [47]. This is an indication of contestation of hegemonic masculinity [33] and hegemonic femininity [24,41,73]. These findings may be a reflection of norms shifting–which corroborates with findings from the 2016 Uganda Demographic and Health Survey, that show a decrease in acceptance of violence from 77% to 49% of women and from 64% to 41% of men between 2000/2001 and 2016 [74]. Our findings also show a positive relationship between education and contestation of violence—likelihood to justify physical violence was less likely among respondents with primary, secondary and tertiary education than those with no education [72]. This suggests that education is likely to play a positive role in shaping positive femininities and masculinities that are less accepting and tolerant to physical violence against women.

While previous studies [69–71] indicate that reproductive rights including conjugal rights are rooted in religious believes, our results show that irrespective of one’s religious belief, people who support the notion that men should have sex with their wives any time were more likely to justify physical violence against women. The study provides support for formal employment as it was associated with not justifying physical violence against women. Respondents who were not formally employed were more likely to justify physical violence than their counterparts who were in formal employment [22,23]. This suggests that interventions that seek to empower communities and support women to access employment and livelihoods may have the potential to transform attitudes towards acceptance of physical violence.

Our study suggests that those who held attitudes favoring the use of violence against women were more likely to justify physical violence. For example, respondents who agreed that it is okay for a man to control his partner’s movements, it is okay for a man to have sex with his wife anytime, alcohol is the main reason for violence against women, men need sex more than women were more likely to justify physical violence. These results point to acceptance of hegemonic masculinity and hegemonic femininity tendencies that are common in patriarchal contexts particularly in Sub-Saharan Africa [24,41,59,73,75]. The findings demonstrate that those who responded in the direction of contestation of beliefs related to violence against women were less likely to justify or condone physical violence. For example, tolerance of physical violence was less among respondents who agreed that: violence is not the only way to deal with disagreements; it is possible for men to stop violence and it is acceptable for a woman to ask her partner to use a condom. Moreover, the distribution of our results–where nearly all respondents agreed that violence is not the only way to solve disagreements–implies that there are alternatives to resolving problems violently. These findings support the adoption of positive masculinities and positive femininities as opposed to hegemonic masculinity and hegemonic femininity [24,41]. The findings on factors that justify the use of IPV indicate that social norm change programmes that target harmful social norms is important. Such programs can support and nurture the positive tendencies towards non-justification of physical violence.

## Conclusions

Accepting attitudes and beliefs towards physical violence are pervasive and common in Uganda. These are sustained by the bedrock of harmful social norms that perpetuate physical violence against women. Our study shows that although justification of physical violence is still prevalent, there are people who contest attitudes tolerating the use of violence and justification for violence. Being educated, married, in formal employment is positively associated with contestation of accepting attitudes towards physical violence against women. Respondents who disagreed with accepting attitudes or beliefs related to physical violence tend to reject justification of physical violence against women. These findings suggest that increasing employment or livelihood or economic empowerment and education particularly targeting women is a protective factor and contributes to contestation of gender inequitable attitudes or beliefs that justify physical violence against women particularly in patriarchal contexts in Uganda. Given that justification of physical violence is driven and sustained by harmful social and gender norms, increasing investment in social norms change programmes has potential to contribute to strengthening contestation of tolerance of physical violence among men and women in Uganda.

### Study limitations and challenges

There are four main limitations associated with this study. First, while every effort is ensured to aim for representativeness, immobile respondents (those who stayed at home for the entire period of the survey was conducted), might not be well represented. Nonetheless, the data used in this study provided an excellent snapshot of what is happening in the community. Second, the effect of alcohol on influencing physical violence may vary depending on the quantities or type of alcohol consumed. However, this study did not investigate the effect of the quantities or type of alcohol consumption on physical violence. Third, we were unable to fully address endogeneity by controlling for all variables that may explain justification of physical violence–which could lead to inconsistent conclusions [76]. Endogeneity occurs when some independent variables are correlated with the error term or when there is correlation between one or more independent variables [77]. While endogeneity is likely to affect results, previous work [78,79] has alluded to the fact that endogeneity cannot be solved completely since the regression models can only capture unobserved heterogeneity that is independent of the variables considered in the model. For example, income may affect employment or perception to violence. Similarly, results may be affected by geographical or cultural factors in the study districts. Finally, basing on the distribution, it is important to note that some attitude indicators used in this paper such as ‘violence is not the only way to deal with disagreements’ may not be the most suitable to explain justification of physical violence against women.

### Recommendations for further studies

We recommend further studies to explore the alternatives to violence against women that are emerging in the communities in resolving disagreements among intimate partners.

## Supporting information

S1 File (DOCX)

## Abbreviations

CEDOVIP: Centre for Domestic Violence Prevention
EU: European Union
GBV: Gender Based Violence
LSHTM: London School of Hygiene and Tropical Medicine
SASA!: Start, Awareness, Support and Action
SDA: Seventh Day Adventist
SSA: Sub-Saharan Africa
UBOS: Uganda Bureau of Statistics
UN: United Nations
UNDP: United Nations Development Program
