# Supplementary material for: An examination of physical violence against women and its justification in development settings in Uganda
Source: PLoS One. 2021 Sep 29;16(9):e0255281. doi: 10.1371/journal.pone.0255281 (PMC8480831; doi:10.1371/journal.pone.0255281)
Supplement: S1 File — (DOCX) [file pone.0255281.s001.docx]

COMMUNITY ASSESSMENT SURVEY

Hello, my name is _________________ and I am working with _____________ to help them learn about your community. If you don’t mind, I would like to ask you a few questions about your thoughts on men and women’s health, relationships and what happens in families in your community. This should not take much of your time, and you can choose to stop the interview at any time, or to skip any questions if you like. Your responses are confidential, and your name will not be written down. We will use the information that you provide to plan activities, and to see how well we are doing in our work.

01. Do you have any questions? YES NO

02. Are you happy to proceed with the interview? YES NO

(IF NO, THANK AND SAMPLE SOMEONE ELSE)

**SECTION 1: ABOUT THE RESPONDENT**

|  | Record the date of the interview | DAY [ ][ ]MONTH [ ][ ] YEAR [2][0][1 ][9] |
| --- | --- | --- |
|  | Record the location of the interview | COMMUNITY/VILLAGE...................................... |
| 101 | Record the respondent’s gender | FEMALE……………………..……………............... 1  MALE………………………………….................... 0  NON- BINARY……………….………………........ 3 |
| 102 | Have you lived in this community / village for at least 1 year? | YES…………………………………………………….0  NO…………………………………………..……….1 (IF NO, THANK AND SAMPLE SOMEONE ELSE) |
| 103 | How old are you? | [ ][ ] (IF LESS THAN 18, THANK AND SAMPLE SOMEONE ELSE) |
| 104 | What is your marital status? | SINGLE ………………….………………................ 0 |
|  |  | MARRIED…………….........................………….. 1 |
|  |  | WIDOWED…………………….....…….................2 |
|  |  | CO-HABITING ..…............................................3 |
|  |  | DIVORCED …………………………..………........ 4 |
|  |  | REFUSE TO ANSWER……………………………….9 |
| 105 | What is your level of education? | NO FORMAL EDUCATION………………............0  COMPLETED PRIMARY EDUCATION…..……… 1  COMPLETED SECONDARY EDUCATION………...2  DIPLOMA HOLDER …........………………........... 3  UNIVERSITY DEGREE…………………………….... 4  OTHER…….............................…………….........  REFUSE TO ANSWER……………………………….9 |
| 106 | What is your religious affiliation? | NO RELIGIOUS AFFILIATION………………........ 0  CATHOLIC…..….................………..................... 1  BORN AGAIN…..............…………..................... 2  PROTESTANT…..........……………...................... 3 BUDDHIST....................................................... ..5  TRADITIONAL/ANIMIST.................................... 6  OTHER............................................................... 7  REFUSE TO ANSWER...........................................9 |
| 107 | Have you been formally employed in the last 3 months? | Yes………………………………………………………0  No…………….……………………….……………….1  REFUSE TO ANSWER.......................................... 9 |

| **Section 2: What we KNOW**  **In this community and elsewhere, people have different ideas about relationships, about families and what is acceptable behavior for men and women in the home. In these questions, we’d like to learn what you think about some of these issues. I am going to read some statements; can you please tell me if you agree or disagree with them? There are no right or wrong answers, so please answer honestly.** | | | |
| --- | --- | --- | --- |
| 201a | **(St)** Everyone can choose to use their power positively or negatively. | AGREE ……………………………………………...  DISAGREE …………………..……………………...  REFUSE TO ANSWER..…………………………… | 0  1  9 |
| 201b | **(Aw)** It is a husband’s role to decide whether or not his wife can work outside the home | AGREE ……………………………………………...  DISAGREE …………………..……………………...  REFUSE TO ANSWER..…………………………… | 0  1  9 |
| 201c | **(Aw)** Violence against women has negative consequences not just for women, but for men as well. | AGREE ……………………………………………...  DISAGREE …………………..……………………...  REFUSE TO ANSWER..…………………………… | 0  1  9 |
| 201d | **(Aw)** It is okay for a man to control his wife’s movements | AGREE ……………………………………………...  DISAGREE …………………..……………………...  REFUSE TO ANSWER..…………………………… | 0  1  9 |
| 201e | **(Aw)** It is fine for a married man to have sex with his wife whenever he wants, even if she does not want to. | AGREE ……………………………………………...  DISAGREE …………………..……………………...  REFUSE TO ANSWER..…………………………… | 0  1  9 |
| 201f | **(Aw)** Men’s power over women is the reason why violence against women happens. | AGREE ……………………………………………...  DISAGREE …………………..……………………...  REFUSE TO ANSWER..…………………………… | 0  1  9 |
| 201g | **(Su)** Violence is not the only way to deal with disagreements or problems in a marriage or relationship – there are alternatives. | AGREE ……………………………………………...  DISAGREE …………………..……………………...  REFUSE TO ANSWER..…………………………… | 0  1  9 |
| 201h | **(Ac)** It is possible for men to stop using violence. | AGREE ……………………………………………...  DISAGREE …………………..……………………...  REFUSE TO ANSWER..…………………………… | 0  1  9 |
| 201i | There is a law that protects women and girls against domestic violence? | AGREE ……………………………………………...  DISAGREE …………………..……………………...  REFUSE TO ANSWER..…………………………… | 0  1  9 |
| 201j | Cases of rape and defilement should be reported within 72 hours | AGREE ……………………………………………...  DISAGREE …………………..……………………...  REFUSE TO ANSWER..…………………………… | 0  1  9 |

| **Section 3: What we FEEL**  **Thank you very much. This next section is about what you think about common issues that come up in relationships between women and men as we are interested in learning your opinion. Please answer yes or no and remember, there are no right or wrong answers.** | | |
| --- | --- | --- |
| 301a | **(St) Do you believe that a woman should tolerate violence from her husband to keep her family together?** | **YES……………………………….………….0**  **NO…………………………………………..1**  **REFUSE TO ANSWER……………………..9** |
| 301b | **(Aw) Do you believe that women are sometimes to blame for violence against them?** | **YES……………………………….………….0**  **NO…………………………………………..1**  **REFUSE TO ANSWER……………………..9** |
| 301c | **(Aw) If a married woman has been beaten by her husband, do you believe it is okay for her to tell others?** | **YES……………………………….………….0**  **NO…………………………………………..1**  **REFUSE TO ANSWER……………………..9** |
| 301d | **(Aw) Do you believe that a non-violent relationship benefits both women and men?** | **YES……………………………….………….0**  **NO…………………………………………..1**  **REFUSE TO ANSWER……………………..9** |
| 301e | **(Su) Do you believe it is acceptable for a married woman to ask her husband to use a condom?** | **YES……………………………….………….0**  **NO…………………………………………..1**  **REFUSE TO ANSWER……………………..9** |
| 301f | **(Su) If a husband is verbally abusing his wife, do you believe others outside the couple should intervene?** | **YES……………………………….………….0**  **NO…………………………………………..1**  **REFUSE TO ANSWER……………………..9** |
| 301g | **(Su) Do you feel you are able to make your relationship healthy and non-violent?** | **YES……………………………….………….0**  **NO…………………………………………..1**  **REFUSE TO ANSWER……………………..9** |
| **301h** | **(Su) Do you believe consent before sex is necessary in a marriage or long-term relationship.** | **YES……………………………….………….0**  **NO…………………………………………..1**  **REFUSE TO ANSWER……………………..9** |
| **301i** | **(Su) If a husband is beating his wife, should others outside the couple intervene?** | **YES……………………………….………….0**  **NO…………………………………………..1**  **REFUSE TO ANSWER……………………..9** |

| **301f** | **(Su) If a husband is verbally abusing his wife, do you believe others outside the couple should intervene?** | **YES……………………………….………….0**  **NO…………………………………………..1**  **REFUSE TO ANSWER……………………..9** |
| --- | --- | --- |
| **301g** | **(Su) Do you feel you are able to make your relationship healthy and non-violent?** | **YES……………………………….………….0**  **NO…………………………………………..1**  **REFUSE TO ANSWER……………………..9** |
| 301h | **(Su)** Do you believe consent before sex is necessary in a marriage or long-term relationship. | YES……………………………….………….0  NO…………………………………………..1  REFUSE TO ANSWER……………………..9 |
| 301i | **(Su)** If a husband is beating his wife, should others outside the couple intervene? | YES……………………………….………….0  NO…………………………………………..1  REFUSE TO ANSWER……………………..9 |
| **Q302: Partners sometimes have disagreements that end up in physical fights. People have different ideas about what is acceptable behavior for men and women in these situations. In your opinion, does a man have a good reason to hit his partner if….** | | |
| 302a | Burns the food | YES……………………………….………….0  NO…………………………………………..1  REFUSE TO ANSWER……………………..9 |
| 302b | She argues with him? | YES……………………………….………….0  NO…………………………………………..1  REFUSE TO ANSWER……………………..9 |
| 302c | She goes out without telling him? | YES……………………………….………….0  NO…………………………………………..1  REFUSE TO ANSWER……………………..9 |
| 302d | She neglects taking care of the children? | YES……………………………….………….0  NO…………………………………………..1  REFUSE TO ANSWER……………………..9 |
| 302e | She refuses to have sexual intercourse with him? | YES……………………………….………….0  NO…………………………………………..1  REFUSE TO ANSWER……………………..9 |

| **What Do You Feel About Your Community?**  **Thank you very much. This next section is about what people feel about their community right now, and we are interested in learning your opinion. Please answer yes or no and remember, there are no right or wrong answers.** | | |
| --- | --- | --- |
| 303a | **(St)** Do you feel you have the power within you to help bring positive change to your community? | YES……………………………….………….0  NO…………………………………………..1  REFUSE TO ANSWER……………………..9 |
| 303b | **(Aw)** Would you say that most members of your community see violence against women as a problem that they want to address? | YES……………………………….………….0  NO…………………………………………..1  REFUSE TO ANSWER……………………..9 |
| 303c | **(Su)** Would you say that in general women and men feel safe to talk about cases of violence in this community? | YES……………………………….………….0  NO…………………………………………..1  REFUSE TO ANSWER……………………..9 |
| 303d | **(Su)** Would you say that in general women in this community feel safe to report their cases of violence in this community? | YES……………………………….………….0  NO…………………………………………..1  REFUSE TO ANSWER……………………..9 |
| 303e | **(Su)** Do you believe it is everyone’s responsibility to help  prevent violence against women in your community? | YES……………………………….………….0  NO…………………………………………..1  REFUSE TO ANSWER……………………..9 |
| 303f | **(Ac)** Do you feel that relationships between women and men have generally become less violent in your community? | YES……………………………….………….0  NO…………………………………………..1  REFUSE TO ANSWER……………………..9 |

| **Section 4: What We Do**  **In the next few questions, I am going to ask you about some common situations that happen in communities. We would like to know what you think about them. Please answer yes or no, there are no wrong answers, please be honest.** | | |
| --- | --- | --- |
| **401a** | **(St)** In the last six months, have you thought much about how you and those around you use power in your personal relationships? | YES……………………………….………….0  NO…………………………………………..1  REFUSE TO ANSWER……………………..9 |
| **401b** | **(Aw)** In the last six months, have you had discussions with anyone about the benefits of changing the way men and women use their power in this community? | YES……………………………….………….0  NO…………………………………………..1  REFUSE TO ANSWER……………………..9 |
| **401c** | **(Su)** In the last six months, have you tried any new ways to balance power in your own relationship?  ** Please select Doesn’t Apply for unpartnered participants* | YES……………………………….………….0  NO…………………………………………..1  DOESN’T APPLY*………………………… 8  REFUSE TO ANSWER…………………. 9 |
| **401d** | **(Su)** In the last six months, did your partner make most of the decisions about when you could have sex?  ** Please select Doesn’t Apply for unpartnered participants* | YES……………………………….………….0  NO…………………………………………..1  REFUSE TO ANSWER……………………..9 |
| **401e** | **(Su)** In the last six months, have you offered support to a woman who you knew was experiencing violence from her husband or boyfriend? | YES……………………………….………….….0  NO……………………………………………...1  Don’t know anyone experiencing violence...8  REFUSE TO ANSWER…………………………9 |
| **401f** | **(Ac)** In the last six months, have you spoken out or taken any action to prevent violence in your community? | YES……………………………….………….0  NO…………………………………………..1  REFUSE TO ANSWER……………………..9 |
| **401g** | Women can easily report cases of violence to the police. | YES……………………………….………….0  NO…………………………………………..1  REFUSE TO ANSWER……………………..9 |

| **Section 5: Exposure to SASA!**  **Thank you so much, we are almost finished. These last questions are about what you see in your community about violence prevention. Please answer yes or no.** | | |
| --- | --- | --- |
| 501 | In the last six months, have you seen people in your community doing something to prevent violence against women? | YES…………………………………..…… 0  NO………………………………………… 1  REFUSE TO ANSWER…………………… 9 |
| 502 | Have you heard of a program called SASA! | YES……………………………….………….0  NO…………………………………………..1  REFUSE TO ANSWER……………………..9 |
| 502a | If yes, do you know a woman or a man who leads SASA! activities in your community?  **If* ***has not*** *heard of* SASA!*, please select Doesn’t Apply* | YES……………………………….………….0  NO…………………………………………..1  REFUSE TO ANSWER……………………..9 |
| 503 | 502b- [If has heard of SASA!, ‘YES’ to Q40]:  Have you seen any SASA! materials such as posters, comics, picture cards, card games, information sheets?  SHOW EXAMPLES OF SASA! MATERIALS  **If* ***has not*** *heard of* SASA!*, please select Doesn’t Apply* | YES…………………………………..…… 0  NO……………………………………… 1  Doesn’t Apply*………………………... 8  REFUSE TO ANSWER………………… 9 |
|  | [If had not heard of SASA!, ‘NO’ to Q40]:  Have you seen other materials about violence against women and relationships between men and women (e.g. posters, comics, brochures, games, information sheets)?  **If* ***has*** *heard of* SASA!*, please select Doesn’t Apply* | YES…………………………………..…… 0  NO……………………………………… 1  Doesn’t Apply*………………………. .. 8  REFUSE TO ANSWER……………… ..9 |
| 504 | In the last six months, have you participated in any activity about safe and healthy relationship? | YES……………………………….………….0  NO…………………………………………..1  REFUSE TO ANSWER……………………..9 |
|  | 504b. If yes, was it a SASA! activity?  **If has not participated, please select Doesn’t Apply* | YES…………………………………..…… 0  NO………………………………………… 1  Doesn’t Apply*…………………………….8  REFUSE TO ANSWER……………………..9 |
| 504c | How many times have you participated in one of these activities?  **If has not participated, please select Doesn’t Apply* | ONCE……………………………………. 1  TWICE…………………………………… 2  MORE THAN TWICE……………… ………3  Doesn’t Apply*……………………………….8  REFUSE TO ANSWER………………………..9 |
| 504d | Do you feel like SASA! is helping make your community safer for women?  **If has not participated, please select Doesn’t Apply* | YES…………………………………..…… 0  NO………………………………………… 1  Doesn’t Apply*…………………………… 8  REFUSE TO ANSWER…………………… 9 |

|  | **SECTION 6. Sexual Reproductive Health Rights**  **In this section I am going to ask you about sexual reproductive health and some common beliefs and practices around sexuality and reproduction especially for women and girls. In these questions, we’d like to learn what you think about some of these issues. I am going to read some statements; can you please tell me if you agree or disagree with them? There are no right or wrong answers, so please answer honestly.** | | |  |
| --- | --- | --- | --- | --- |
|  | **Knowledge** |  |  |  |
| 601a | Using heroin or opioids can prevent pregnancy | AGREE  DISAGREE  REFUSE TO ANSWER | 0  1  9 |  |
| 601b | If my partner is using heroin or opioids this can prevent pregnancy | AGREE  DISAGREE  REFUSE TO ANSWER | 0  1  9 |  |
| 601c | Consistently and correctly using condoms is an effective method to prevent pregnancy | AGREE  DISAGREE  REFUSE TO ANSWER | 0  1  9 |  |
| 601d | It is not easy for a young women (under 20 years) to get pregnant | AGREE  DISAGREE  REFUSE TO ANSWER | 0  1  9 |  |
| 601e | Douching/cleaning vagina after sex intercourse can prevent pregnancy | AGREE  DISAGREE  REFUSE TO ANSWER | 0  1  9 |  |
| 601f | If the man pulls his penis out of my vagina before ejaculation, I will have no risk of getting pregnant | AGREE  DISAGREE  REFUSE TO ANSWER | 0  1  9 |  |
| 601g | Alcohol is the main cause of Violence against women and girls | AGREE  DISAGREE  REFUSE TO ANSWER | 0  1  9 |  |
|  | **Attitudes** |  |  |  |
| 602a | If a man told his friends that he makes decisions jointly with his partner, would his friends respect him? | YES  NO  Doesn’t apply  REFUSE TO ANSWER | 0  1  8  9 |  |
| 602b | Do you think that it's a man's role to decide whether or not his partner can work outside the home? | YES  NO  Doesn’t apply  REFUSE TO ANSWER | 0  1  8  9 |  |
| 602c | In your opinion, is it true that a married man needs other women? | YES  NO  Doesn’t apply  REFUSE TO ANSWER | 0  1  8  9 |  |
| 602d | In your opinion, is it true that a married man needs other women, even if things with his wife are fine? | YES  NO  Doesn’t apply  REFUSE TO ANSWER | 0  1  8  9 |  |
| 602e | Is it acceptable for a woman to ask her partner to use a condom? | YES  NO  Doesn’t apply  REFUSE TO ANSWER | 0  1  8  9 |  |
| 602f | In your opinion, is it acceptable if a woman refuses to have sex with her partner if she doesn't feel like it? | YES  NO  Doesn’t apply  REFUSE TO ANSWER | 0  1  8  9 |  |
| 602g | All women/girls have a right to access contraceptives/family planning services | AGREE  DISAGREE  REFUSE TO ANSWER | 0  1  9 |  |
| 602h | A girl who is sexually active can use contraceptives to prevent unwanted pregnancy. | AGREE  DISAGREE  REFUSE | 0  1  9 |  |
| 602i | Unmarried women/girls should have access to contraception/family planning services | AGREE  DISAGREE  REFUSE TO ANSWER | 0  1  9 |  |
| 602j | Married women/girls should have access to contraception/family planning service | AGREE  DISAGREE  REFUSE TO ANSWER | 0  1  9 |  |
| 602k | Giving unmarried women/girls access to contraceptives makes them promiscuous | AGREE  DISAGREE  REFUSE TO ANSWER | 0  1  9 |  |
| 602l | Women know where to access contraceptives for prevention of pregnancy. | AGREE  DISAGREE  REFUSE TO ANSWER | 0  1  9 |  |
| 602m | A woman can decide on how to use their finances without asking their husband. | AGREE  DISAGREE  REFUSE TO ANSWER | 0 |  |
| 602n | I would like contraceptives/family planning services to be available to women/girls in my community | AGREE  DISAGREE  REFUSE TO ANSWER | 0  1  9 |  |
|  | **Please indicate if you totally agree, partially agree, or disagree with the following statements: READ EACH STATEMENT ALOUD** | | |  |
| 603a | A woman has a right to refuse sex. | AGREE  DISAGREE  REFUSE TO ANSWER | 0  1  9 |  |
| 603b | A woman can decide on the number of children she wants and when to have them without asking her husband | AGREE  DISAGREE  REFUSE TO ANSWER |  |  |
| 603c | Men are always ready to have sex. | AGREE  DISAGREE  REFUSE TO ANSWER | 0  1  9 |  |
| 603d | Men need sex more than women do. | AGREE  DISAGREE  REFUSE TO ANSWER | 0  1  9 |  |
| 603e | It is safe for women to use contraceptives. | AGREE  DISAGREE  REFUSE TO ANSWER | 0  1  9 |  |
| 603f | It is solely a woman’s responsibility to avoid getting pregnant. | AGREE  DISAGREE  REFUSE TO ANSWER | 0  1  9 |  |
| 603f | Women who carry condoms are promiscuous. | AGREE  DISAGREE  REFUSE TO ANSWER | 0  1  9 |  |
| 604a | It disgusts me when I see a man acting like a woman | AGREE  DISAGREE  REFUSE TO ANSWER | 0  1  9 |  |
| 604b | Only when a woman gives birth to a child is she a real woman | AGREE  DISAGREE  REFUSE TO ANSWER | 0  1  9 |  |
| 604c | Only when a man has a child will he be a respected member of his clan. | AGREE  DISAGREE  REFUSE TO ANSWER | 0  1  9 |  |
| 604d | A man and a woman should decide together what type of contraceptive to use. | AGREE  DISAGREE  REFUSE TO ANSWER | 0  1  9 |  |
| 604e | An ideal married couple will produce a child in their first year of marriage | AGREE  DISAGREE  REFUSE TO ANSWER | 0  1  9 |  |
| 604f | Men should be offended if their wives ask them to use a condom. | AGREE  DISAGREE  REFUSE TO ANSWER | 0  1  9 |  |
| 604g | All contraceptives have negative effects on women’s lives and are responsible for cancers among women | AGREE  DISAGREE  REFUSE TO ANSWER | 0  1  9 |  |
| 604h | It is not right and acceptable for a woman to ask her partner to use a condom, she will be regarded a harlot? | AGREE  DISAGREE  REFUSE TO ANSWER | 0  1  9 |  |
| 604i | Sex is not safe until it suggested and approved by a man | AGREE  DISAGREE  REFUSE TO ANSWER | 0  1  9 |  |
| 604j | It is not good for girls to know about their sexuality and other sexual matters because they will get spoilt | AGREE  DISAGREE  REFUSE TO ANSWER | 0  1  9 |  |
|  | | **Practice: IF RESPONDENT HAS NEVER BEEN MARRIED OR LIVED WITH A PARTNER, TICK THE BOX AND SKIP** | | |
|  |  | **IF RESPONDENT HAS EVER BEEN MARRIED OR LIVED WITH A PARTNER, CONTINUE WITH THIS MODULE** | | |
|  |  |  | | |
| 605 | | Have you been married or living with a partner as if married within the past 12 months? | YES | 1 |
|  |  |  | NO | 0 |
| 606 | | In the last 12 months, has your partner had more say than you do about important decisions that affect your relationship/family? | YES | 1 |
|  |  |  | NO | 0 |
| 607 | | During the last 12 months, has your partner made most of the decisions about your own health care? | YES | 1 |
|  |  |  | NO | 0 |
| 608 | | During the last 12 months, has your partner made most of the decisions about whether or not to use family planning and what method to use? | YES | 1 |
|  |  |  | NO | 0 |
| 609: | | If a woman uses a family planning method without her husband knowing, and he finds out, she deserves to be beaten | YES | 1 |
| 610: | | Would you say that using contraception is mainly your decision, mainly your husband’s/wife’s or live-in partner’s decision, or did you both decide together? | YES | 1 |
| 611: | | In your household are you able to make final and binding decisions about the use of contraceptives? | NO | 0 |
| 612: | | Who makes the most money, you, your partner, or both of you equally? | You  Partner  Same  NA | 1  2  3  88 |
| 609: | | If a woman uses a family planning method without her husband knowing, and he finds out, she deserves to be beaten | YES | 1 |
|  | | Now I would like to ask you some questions about how decisions are made in your relationship. Do you (1), your partner (2) , both you and your partner (3), or someone else (88) make the following decisions? |  |  |
| 614a | | such as buying a cow, buying a bicycle etc. |  |  |
| 614b | | Making purchases for daily household needs (such as buying food, buying cooking oil etc.) |  |  |
| 614c | | Spending household resources on your healthcare |  |  |
| 614d | | Whether or not to have sex |  |  |
| 616 | | In our community only health care workers are preferred to be the primary source of information on SRH issues | AGREE  DISAGREE  REFUSE TO ANSWER | 0  1  9 |
| 617 | | In our community most people agree with "sex education will lead to more sexual behavior” and so they fight it | AGREE  DISAGREE  REFUSE TO ANSWER | 0  1  9 |
| 619 | | In my community parents will punish a child if they know that she/he went to seek any sex related information or services from any where | AGREE  DISAGREE  REFUSE TO ANSWER | 0  1  9 |
| 619 | | In my community the elders will punish unmarried women/girls trying to access to contraceptives because makes them promiscuous | AGREE  DISAGREE  REFUSE TO ANSWER | 0  1  9 |

|  | **Section 7: Early Marriage**  **Am going to ask you some questions about early marriages. People have different ideas about early marriages and I would like to know what you think. There is no right or wrong answers. For the statements below please tell me whether you agree or disagree with them related to prudential reasons, personal normative beliefs, and social expectations regarding child marriage** | | |
| --- | --- | --- | --- |
| 701 | Do you know what the legal age for marriage is (for girls/boys)? | YES  NO  Doesn’t apply  REFUSE TO ANSWER | 0  1  8  9 |
| 701b | If yes what is the legal age of marriage |  | |
| 702 | What is the appropriate age for girls to marry in this community? |  | |
| 703 | What is the appropriate age for boys to marry in this community? |  | |
| 704 | Do some girls marry later or not at all? | YES  NO  Doesn’t apply  REFUSE TO ANSWER | 0  1  8  9 |
| 705 | How are girls who marry later perceived in your community |  | |
| 706 | Are there specific people/groups who actively work to maintain girls marrying at an early age | YES  NO  Doesn’t apply  REFUSE TO ANSWER | 0  1  8  9 |
| 708 | If yes, Who are these people/groups? | 1 = Mentioned 2 = Not Mentioned | |
| 708a | Girls themselves |  | |
| 708b | Uncles/Aunties |  | |
| 708c | Grandparents |  | |
| 708d | Father |  | |
| 708e | Mother |  | |
| 708f | Sisters |  | |
| 708g | Brothers |  | |
| 708h | A village elder |  | |
| 708i | Friends/peers |  | |
| 708k | Other |  | |
|  |  |  | |
|  |  |  | |
| 710 | Are there specific groups working to move the desirable age of marriage for girls? | YES  NO  Doesn’t apply  REFUSE TO ANSWER | 0  1  8  9 |
| 710b | If yes Who are they? |  | |
| 711 | What are the incentives for parents to marry their girls at an early age? |  | |
| 712 | What are the incentives for parents to marry their girls at later age? |  | |
| 713 | What are the incentives for girls to marry at an early age? |  | |
| 174 | What are the incentives for girls to marry at a later age? |  | |
| 715 | If you want your daughter to get married early and she refuses, what happens |  | |
| 716 | If you and your spouse disagree about the age at which your daughter marries, what happens? |  | |
| 717 | If you are not willing to marry your daughter off early, what happens? |  | |
| 718 | If the expected age of marriage differs from the legal age, how do people decide what to do? |  | |
| 719 | Is it common for girls in this community to have children before they are married? | YES  NO  Doesn’t apply  REFUSE TO ANSWER | 0  1  8  9 |
| 720 | What effect does having a child have on an unmarried girl’s life |  | |
| 721 | How are girls having babies when not married viewed |  | |
| 722 | What do you do if your son/daughter has a child without being married |  | |
| 723 | What challenges does having a baby when not married create for the girl |  | |
| 724 | Are young girls allowed to get contraceptives or access other family planning services? | Yes 1  No 0 | |
| 725 | Who makes the decision for girls to get contraceptives or access other family planning services? | Yes 1  No 0 | |
|  | **For the statements below please tell me whether you agree or disagree with them related to prudential reasons, personal normative beliefs, and social expectations regarding child marriage** | | |
| 730a | Most girls in this community marry before the age of 18 years | AGREE  DISAGREE  REFUSE TO ANSWER | 0  1  9 |
| 730b | Most people in this community expect girls to marry before the age of 18 years | AGREE  DISAGREE  REFUSE TO ANSWER | 0  1  9 |
| 730c | If I do not ensure my daughters and/or nieces are married early, my family will not be respected in the community | AGREE  DISAGREE  REFUSE TO ANSWER | 0  1  9 |
| 730d | Marrying girls at a young age can help provide them security | AGREE  DISAGREE  REFUSE TO ANSWER | 0  1  9 |
| 730e | Marrying girls young can help prevent sexual violence, assault, and harassment | AGREE  DISAGREE  REFUSE TO ANSWER | 0  1  9 |
| 730f | It is wrong to marry a girl before the age of 18 | AGREE  DISAGREE  REFUSE TO ANSWER | 0  1  9 |
| 730g | Marriage of girls under 18 y sometimes happens for financial reasons | AGREE  DISAGREE  REFUSE TO ANSWER | 0  1  9 |
| 730h | Marriage of girls under 18 y mostly happens because there is a lack of education and job opportunities | AGREE  DISAGREE  REFUSE TO ANSWER | 0  1  9 |
| 730i | Marriage of girls under 18 y may happen because of pregnancy in this community | AGREE  DISAGREE  REFUSE TO ANSWER | 0  1  9 |
| 730j | It is acceptable for women/girls to have sex before marriage | AGREE  DISAGREE  REFUSE TO ANSWER | 0  1  9 |
| 730k | It is acceptable for men/boys to have sex before marriage | AGREE  DISAGREE  REFUSE TO ANSWER | 0  1  9 |
| 730l | Unmarried women/girls who get pregnant are naughty | AGREE  DISAGREE  REFUSE TO ANSWER | 0  1  9 |
|  |  |  |  |
|  | **Intersections between marriage and education for girls** |  |  |
| 731 | Should an 18-year-old girl be in school or married?  State some | YES  NO  Doesn’t apply  REFUSE TO ANSWER | 0  1  8  9 |
| 732 | Is it common for girls to be withdrawn from school for marriage? | YES  NO  Doesn’t apply  REFUSE TO ANSWER | 0  1  8  9 |
| 733 | What do you think about married girls going back to school? |  | |
| 734 | Do you have friends or relatives who have had any experience of this? | YES  NO  Doesn’t apply  REFUSE TO ANSWER | 0  1  8  9 |
| 735 | What do you think about unmarried girls with children going back to school? |  | |
| 736 | Are there any special programmes that help married girls or adolescent mothers to continue their schooling? | YES  NO  Doesn’t apply  REFUSE TO ANSWER | 0  1  8  9 |
| 737 | Do you think more educated girls have more problems finding husbands than less educated girls? | YES  NO  Doesn’t apply  REFUSE TO ANSWER | 0  1  8  9 |
| 737b | If yes why |  | |
| 738 | Do you think being more educated makes girls better wives, mothers, daughters-in-law? | YES  NO  Doesn’t apply  REFUSE TO ANSWER | 0  1  8  9 |
| 738b | If yes why |  | |
| 738c | Do you know of any girls/women |  | |
|  | **Laws, programmes, policies and services** |  |  |
| 739 | Are you aware of any laws/policies that prohibit early marriage? | YES  NO  Doesn’t apply  REFUSE TO ANSWER | 0  1  8  9 |
| 739b | If yes please List |  | |
| 740 | Are you aware of any community initiatives that are encouraging girls not to marry early? | YES  NO  Doesn’t apply  REFUSE TO ANSWER | 0  1  8  9 |
| 740b | If yes please List |  | |
| 741 | In your opinion how effective are these programs? | Effective  Less effective  Not effective  DK | 0  1  2  9 |

|  | | **Section 8: Female Genital Mutilation/Cutting: Now I am going to ask you about some traditional practices common in some communities in Uganda. Some of the practices generally relate to FGM in your or other communities you have heard of. Please answer honestly as there no right or wrong answers.** | | | | | | | | | | | |
| --- | --- | --- | --- | --- | --- | --- | --- | --- | --- | --- | --- | --- | --- |
| **NO.** | | **QUESTIONS** | **CODING CATEGORIES** | | | | | | | | |  | |
|  | | Now I would like to ask for your opinion about a cultural practice called female circumcision that sometimes occurs in your community. People have different ideas about female circumcision and I would like to know what you think. There are no right or wrong answers. | | | | | | | | | | | |
| 801 | | Have you ever heard of female circumcision? | | | | YES  NO | | 1  2 | | | |  | |
| 802 | | In some countries, there is a practice in which a girl may have part of her genitals cut. Have you ever heard about this practice? | | | | Yes  No | | 1  2 | | | |  | |
| **803** | | **Attitudes: Please tell me whether you agree with, disagree with, or are not sure about the following statements related to female circumcision: ( READ EACH STATEMENT ALOUD)** | | | | | | | | | | | |
|  | | Female genital cutting has no health consequences | | | **AGREE** | | | | **DISAGREE** | | | | **UNSURE** |
|  | | Female genital cutting prevents promiscuity and reduces HIV infections | | |  | | | |  | | | |  |
|  | | Female genital cutting is a good cultural practice and an identity of the people practicing it | | |  | | | |  | | | |  |
|  | | Female genital cutting is the most effective way to control promiscuity among women | | |  | | | |  | | | |  |
|  | | Most non-mutilated women / girls are likely to remain barren until they are mutilated | | |  | | | |  | | | |  |
|  | | Female circumcision violates human rights | | |  | | | |  | | | |  |
|  | | Female circumcision preserves virginity. | | | 1 | | | | 0 | | | | 98 |
|  | | Female circumcision ensures marital faithfulness. | | | 1 | | | | 0 | | | | 98 |
|  | | Female circumcision prevents prostitution. | | | 1 | | | | 0 | | | | 98 |
|  | | A girl must be circumcised before she can marry. | | | 1 | | | | 0 | | | | 98 |
|  | | A girl must be circumcised in order to become an adult woman | | | 1 | | | | 0 | | | | 98 |
|  | | Female circumcision makes girls clean. | | | 1 | | | | 0 | | | | 98 |
|  | | Female circumcision makes girls beautiful. | | | 1 | | | | 0 | | | | 98 |
|  | | Female circumcision cleanses evil spirits. | | | 1 | | | | 0 | | | | 98 |
|  | | Uncircumcised women are dirty. | | | 1 | | | | 0 | | | | 98 |
|  | | If an uncircumcised woman gives birth, her baby will die. | | | 1 | | | | 0 | | | | 98 |
|  | | Failure to circumcise a girl will bring a curse upon her. | | | 1 | | | | 0 | | | | 98 |
|  | | Failure to circumcise a girl will bring a curse upon the entire community. | | | 1 | | | | 0 | | | | 98 |
|  | | Girls should marry as soon as they are circumcised. | | | 1 | | | | 0 | | | | 98 |
|  | | Female circumcision reduces sexual feelings. | | | 1 | | | | 0 | | | | 98 |
|  | | Female circumcision is a good practice. | | | 1 | | | | 0 | | | | 98 |
|  | | Female circumcision makes genitalia more attractive. | | | 1 | | | | 0 | | | | 98 |
| **804** | | **Please tell me whether you agree with, disagree with, or are not sure about the following statements related to female circumcision: ( READ EACH STATEMENT ALOUD)** | | |  | | | |  | | | |  |
|  | |  | | | **AGREE** | | | | **DISAGREE** | | | | **UNSURE** |
|  | | Uncircumcised girls/women are not allowed to fetch water from | | | 1 | | | | 0 | | | | 98 |
|  | | Uncircumcised girls/women are not allowed to use the communal | | | 1 | | | | 0 | | | | 98 |
|  | | The children of uncircumcised women are not allowed to play with | | | 1 | | | | 0 | | | | 98 |
|  | | Husbands of uncircumcised women ask traditional birth attendants to circumcise them | | | 1 | | | | 0 | | | | 98 |
|  | | In our community for a woman to be respected and married she has to be circumcised. | | | 1 | | | | 0 | | | | 98 |
|  | | None-mutilated women are cursed and are less likely to find a good man to marry | | | 1 | | | | 0 | | | | 98 |
|  | | Non-circumcision for women is responsible for the increased number of women undergoing operations during child delivery | | | 1 | | | | 0 | | | | 98 |
|  | | Failure to circumcise a girl will bring a curse upon the entire community | | | 1 | | | | 0 | | | | 98 |
| **805** | | **PRACTICE Please tell me whether you agree with, disagree with, or are not sure about the following statements related to female circumcision: ( READ EACH STATEMENT ALOUD)** | | | **Most** | | | | **A few** | | | | **None** |
|  | | Would you say that most members of your community consider female genital cutting a problem that should urgently be addressed? | | | 1 | | | | 0 | | | | 98 |
|  | | Would you say that in general most men feel safer to marry a mutilated woman/girl in this community because she is more likely to remain faithful? | | | 1 | | | | 0 | | | | 98 |
|  | | Would you say that in general most women and men feel safe not to talk about the prevention of female genital cutting in this community? | | | 1 | | | | 0 | | | | 98 |
|  | | In this community how many community members believe that if an uncircumcised woman gives birth, her baby will die | | | 1 | | | | 0 | | | | 98 |
| 805e | | For Men only: If you were getting married for the first time today, would you marry an uncircumcised woman? | | | YES  NO  REFUSE TO ANSWER | | | | | | | | 0  1  9 |
| 805f | | Would you encourage your relatives / people in your community who have daughters not to get cut? | | | YES  NO  REFUSE TO ANSWER | | | | | | | | 0  1  9 |
| 805g | | Would you support your son or any other male member of your family to marry only a cut girl? | | | YES  NO  REFUSE TO ANSWER | | | | | | | | 0  1  9 |
| 806 | | Do you intend to have any of your daughters circumcised in the future? | | | Yes | | | | | | | | 1 |
|  |  |  |  |  | No | | | | | | | | 0 |
|  |  |  |  |  | Don't have any daughters | | | | | | | | **2** |
|  |  |  |  |  | Don't know | | | | | | | | 98 |
|  | | Have you heard of any marriages in your community where the woman was not circumcised? | | | Yes | | | | | | | | 1 |
|  |  |  |  |  | No | | | | | | | | 0 |
| 807 | | How many of marriages of uncircumcised women are you aware of in your community--a few, some, most, or all? | | | A few | | | | | | | | 1 |
|  |  |  |  |  | Some | | | | | | | | 2 |
|  |  |  |  |  | Most | | | | | | | | 3 |
|  |  |  |  |  | All | | | | | | | | 4 |
| 808 | | In these marriages in your community where the woman was not circumcised, was the bride price usually higher, lower, or the same as the marriages for circumcised women? | | | Lower for uncircumcised women | | | | | | | | 1 |
|  |  |  |  |  | Same | | | | | | | | 2 |
|  |  |  |  |  | Higher for uncircumcised women | | | | | | | | 3 |
|  |  |  |  |  | Don't know | | | | | | | | 98 |
| 809 | | Do you think that female circumcision should be continued, or should it be stopped? | | | Continued | | | | | | | | 1 |
|  |  |  |  |  | Stopped | | | | | | | | 2 |
|  |  |  |  |  | Depends | | | | | | | | 3 |
|  |  |  |  |  | Don’t know | | | | | | | | 4 |
| 810 | | Do you think that any girl in your community will be cut in the next 12 months? | | | YES  NO  REFUSE TO ANSWER | | | | | | | | 0  1  9 |
| 811 | | Do you think that any girl in your community has been cut in the past 12 months? | | | YES  NO  REFUSE TO ANSWER | | | | | | | | 0  1  9 |
| 812 | | Do you have friends or relatives who have had any experience of this? | | | YES  NO  REFUSE TO ANSWER | | | | | | | |  |
|  | | **Knowledge** | | |  | | | | | | | |  |
| 815 | | Do you believe there are any negative consequences of female circumcision? | | | Yes | | | | | | | | 1 |
|  |  |  |  |  | No | | | | | | | | 2 |
|  |  |  |  |  | Don’t know | | | | | | | | 3 |
| 815_b | | What are the negative consequences of female circumcision?  DO NOT READ ALOUD; CHECK ALL THAT THE RESPONDENT MENTIONS. | | | Pain during the procedure  Bleeding  Shock  Infections (other than HIV)  HIV  Problems urinating  Pain during sex  Genital disfigurement  Reduction of sexual feelings  Psychological trauma  Menstrual problems  Problems giving birth  Fistula  Death  Other  Don't know | | | | | | | | 1  2  3  4  5  6  7  8  9  10  11  12  13  14  15  99 |
| **820** | | **Do you believe that if FGM/C is abandoned** | | | **AGREE** | | | | | **DISAGREE** | | | **DON’T KNOW** |
| A | | Girls would not get married before 18 years? | | | 1 | | | | | 2 | | | 3 |
| B | | Girls would not get pregnant at a young age? | | | 1 | | | | | 2 | | | 3 |
| C | | Girls would not drop out of school? | | | 1 | | | | | 2 | | | 3 |
| D | | Maternal and new born death would reduce? | | | 1 | | | | | 2 | | | 3 |
| E | | Women/girls with fistula would re­duce? | | | 1 | | | | | 2 | | | 3 |
| F | | Women/girls getting infected with HIV would reduce? | | | 1 | | | | | 2 | | | 3 |
| G | | Expenditure on health care to wom­en/girls would reduce? | | | 1 | | | | | 2 | | | 3 |
| **821** | | **In the past 12 months have you** | | | **YES** | | | | | **NO** | | | **REFUSE TO ANSWER** |
| A | | Heard about FGM/C on the radio? | | | 0 | | | | | 1 | | | 9 |
| B | | Seen anything about FGM/C on the television? | | | 0 | | | | | 1 | | | 9 |
| C | | Read about FGM/C in the newspaper/ magazine? | | | 0 | | | | | 1 | | | 9 |
| D | | Received a voice or text message about FGM/C on the phone? | | | 0 | | | | | 1 | | | 9 |
| E | | Heard / seen anything about FGM/C at a community drama / sports event? | | | 0 | | | | | 1 | | | 9 |
| **822: Now, I would like to ask you about the laws related to FGM/C in Uganda. I only wish to know your understanding of the laws; I will not ask any questions about your experiences of or involvement in female circumcision.**  **Please tell me whether the following statements are true or false: READ EACH STATEMENT ALOUD** | | | | | | | | | | | | | |
|  |  | | | **TRUE** | | | **FALSE** | | | | **DON'T KNOW** | | |
| A | Performing female circumcision on someone else is a crime in Uganda. | | | 1 | | | 0 | | | | 98 | | |
| B | It is legal to perform female circumcision on oneself in Uganda. | | | 1 | | | 0 | | | | 98 | | |
| C | Simply attempting to perform female circumcision is a crime in Uganda. | | | 1 | | | 0 | | | | 98 | | |
| D | Counseling someone to undergo female circumcision is a crime | | | 1 | | | 0 | | | | 98 | | |
| E | Participating in an event leading to female circumcision is a crime in Uganda | | | 1 | | | 0 | | | | 98 | | |
| F | If a girl consents to undergo female circumcision, it is not a crime in | | | 1 | | | 0 | | | | 98 | | |
| G | Female circumcision is not a crime in Uganda if your religion requires it. | | | 1 | | | 0 | | | | 98 | | |
| H | Discrimination against uncircumcised women is a crime in Uganda. | | | 1 | | | 0 | | | | 98 | | |
| **824 Options for support**  **In the next few questions, I am going to ask you about some common situations that happen in communities. We would like to know what you think is the best way to address them. Please answer yes or no, there are no wrong answers, please be honest.** | | | | | | | | | | | | | |
| a | | Are you aware of any ongoing initiatives in your community to stop the practice of female circumcision? | | | YES  NO  REFUSE TO ANSWER | | | | | | | | 0  1  9 |
| b | | In the last six months, have you thought much about how you and those around you can prevent female genital cutting? | | | YES  NO  REFUSE TO ANSWER | | | | | | | | 0  1  9 |
| c | | In the last six months, have you had discussions with anyone about the benefits of protecting the rights of women and girls who are not mutilated in this community? | | | YES  NO  REFUSE TO ANSWER | | | | | | | | 0  1  9 |
| d | | Am aware of about the effects of female circumcision and I have shared this information with my neighbor and my friends | | | AGREE  DISAGREE  REFUSE TO ANSWER | | | | | | | | 0  1  9 |
| e | | Everyone in my community is committed to help women/girls who may experience female circumcision | | | AGREE  DISAGREE  REFUSE TO ANSWER | | | | | | | | 0  1  9 |

**END**

Thank you for your time. I really appreciate you talking with me and sharing your thoughts.

Would you like a list of organizations/people who you could talk confidentially with about any of these issues?

(If yes, give referral list. If no, thank again and remind them of the name of your organization in case they are interested in follow up.)

Data entered on____ by:_________ Form ID:___
